# Influenza, Pneumococcal and Herpes Zoster Vaccination Rates in Patients with Autoimmune Inflammatory Rheumatic Diseases

**DOI:** 10.3390/vaccines11040760

**Published:** 2023-03-29

**Authors:** Marco Krasselt, Ulf Wagner, Olga Seifert

**Affiliations:** Medical Clinic III, Endocrinology, Nephrology and Rheumatology, Leipzig University, Liebigstr. 20, 04103 Leipzig, Germany

**Keywords:** vaccination, influenza, streptococcus pneumoniae, COVID-19, rheumatic diseases

## Abstract

Background: Vaccination rates are known to be low in patients with autoimmune inflammatory rheumatic diseases (AIIRD). We therefore aimed to determine current vaccination rates against influenza, Streptococcus pneumoniae and herpes zoster in a cohort of patients with AIIRD in Germany. Methods: Consecutive adult patients with an AIIRD were recruited from our outpatient clinic during their regular consultations. The individual vaccination status regarding influenza, Streptococcus pneumoniae and herpes zoster was obtained by reviewing the vaccination documents. Results: A total of 222 AIIRD patients (mean age 62.9 ± 13.9 years) were included. In total, 68.5% were vaccinated against influenza, 34.7% against Streptococcus pneumoniae and 13.1% against herpes zoster (HZ). The pneumococcal vaccination was outdated in 29.4% of the vaccinated patients. Vaccination rates were significantly higher in patients ≥60 years old (odds ratio (OR) 2.167, 95% confidence interval (CI) 1.213–3.870, *p* = 0.008 for influenza, OR 4.639, 95% CI 2.555–8.422, *p* < 0.0001 for pneumococcal and OR 6.059, 95% CI 1.772–20.712, *p* = 0.001 for HZ vaccination). Ages > 60 years, female sex, glucocorticoid use and influenza vaccination were all independently associated with a pneumococcal vaccination. Regarding influenza vaccination, only a positive pneumococcal vaccination history remained independently associated. In patients with HZ vaccination, glucocorticoid use and a preceding pneumococcal vaccination were independently associated with HZ protection. Conclusions: The frequencies of vaccinations against influenza, Streptococcus pneumoniae and HZ have increased during recent years. While this can be partly explained by continuous efforts in patient education during the outpatient visits, the COVID-19 pandemic might also have contributed. Nevertheless, the persistently high incidence and mortality of these preventable diseases in patients with AIIRDs mandates further efforts to increase vaccination coverage, particularly in SLE patients.

## 1. Introduction

While they are easily preventable by vaccination, influenza and Streptococcus pneumoniae pose a known risk to patients with autoimmune inflammatory rheumatic diseases (AIIRD). Epidemiological studies demonstrated that patients with AIIRDs have a higher risk of acquiring infections with a disproportional increased risk of infection mortality [1,2]. Streptococcus pneumoniae, for example, is the most common cause for community-acquired pneumonia in Europe [3]. With an incidental risk ratio (IRR) of up to 5.0 (95% confidence interval (CI) 4.6–5.4) for invasive pneumococcal disease (IPD) in patients with systemic lupus erythematosus (SLE) and an IRR of 1.2 (95% CI 1.1–1.4) for influenza in patients with rheumatoid arthritis (RA), the incidence for both diseases is increased in AIIRD patients and contributes significantly to their mortality [4,5,6]. Similarly, the incidence of herpes zoster is significantly increased in these patients: while the incidence is 3–5/1.000 person years in the general population, it reaches up to 12/1.000 person-years in RA and up to 91/1.000 person-years in SLE patients, [7]. Compared to healthy controls, humoral immunity against the varicella-zoster virus is qualitatively and quantitatively reduced in RA patients, probably due to immune cell exhaustion and immunosenescence [7,8].

The European League Against Rheumatism (EULAR) therefore recommends vaccination against both influenza and Streptococcus pneumoniae for the majority of patients with rheumatic diseases [9]. Similar recommendations come from the German Standing Committee on Vaccination (STIKO) [10]: vaccination against influenza and Streptococcus pneumoniae is recommended for people of any age with chronic diseases, immunodeficiency or immunosuppressive therapy and for people ≥ 60 years old. The influenza vaccination should be applied every year using a quadrivalent vaccine based on the recommendations of the World Health Organization (WHO). Regarding pneumococcal vaccination, a stepwise approach (13-valent pneumococcal conjugate vaccine (PCV13) followed by the 23-valent pneumococcal polysaccharide vaccine (PPSV23) for the first immunization) is recommended for patients with AIIRDs, while a vaccination with PPSV23 alone should be sufficient for healthy people ≥ 60 years old. A pneumococcal booster should be considered after at least six years using the PPSV23 vaccine only. Furthermore, pneumococcal vaccination is recommended for all infants ≥ two months old before the age of two. Primarily, PCV13 should be used in a 2 + 1 scheme (baseline + eight weeks + six months) [10]. Based on the EULAR recommendation, a herpes zoster vaccination “may be considered in high-risk patients” [9]. Importantly, this recommendation primarily addresses the live vaccine. In contrast to the EULAR recommendations, the German STIKO clearly recommends a herpes zoster vaccination with the subunit vaccine for both RA and SLE patients ≥ 50 years old as well as healthy people ≥ 60 years old. Booster vaccinations are not recommended as well as the live vaccine [10].

As we know from earlier investigations on vaccination coverage, vaccination rates for basic vaccines are generally low in AIIRD patients [1,2,11,12].

Therefore, patients’ individual vaccination documents are rigorously and regularly reviewed in our outpatient clinic, and individual vaccination recommendations for the attending GP have been made. During the summer of 2021, we conducted a cross-sectional study to evaluate the current vaccination rates regarding influenza, Streptococcus pneumoniae and herpes zoster.

## 2. Materials and Methods

In total, 222 consecutive adult patients with an autoimmune inflammatory rheumatic disease (AIIRD) were recruited from our outpatient clinic at the Leipzig University Hospital in Germany during their regular consultations between June and August 2021. Patients were asked for their vaccination status regarding influenza, Streptococcus pneumoniae and herpes zoster. No vaccinations were administered in our outpatient clinic. Any reported vaccination was double-checked by reviewing the individual vaccination cards. Patients’ characteristics were obtained from the medical record and are shown in Table 1.

### Biostatistical Analysis

To describe continuous data, mean and standard deviation were used, categorical data were described with absolute and/or relative frequencies. To compare the frequencies of categorical variables, a Chi square test was performed. Odds ratios are given as point estimates with a 95% confidence interval. For multivariate analysis, binary logistic regression was used and all covariates from the bivariate analysis were entered (fully adjusted model). A significant statistical difference was assumed when the *p* value was below 0.05. All analyses were two-tailed and conducted using SPSS Statistics Version 24 for Mac (IBM Corp., Armonk, NY, USA).

Informed consent was obtained from all patients participating in the study. The design of the study was approved by the local ethics committee of the University of Leipzig (282/21-ek).

## 3. Results

### 3.1. Study Population

In total, 222 patients with various rheumatic diseases (mean age 62.9 ± 13.9 years, 68.9% female, Table 1) were included. The largest group of recruited patients (44%) had rheumatoid arthritis (RA), 25% had spondyloarthritis (SpA), 18.5% had systemic lupus erythematosus (SLE), and 13.1% had been diagnosed with other AIIRDs (see Table 1 for details).

### 3.2. Vaccination Coverage

In total, 152 of 222 patients (68.5%) were vaccinated against influenza within the last year, 109 of 222 patients (49.1%) had had a pneumococcal vaccination and 29 of 222 (13.1%) patients were vaccinated against herpes zoster (Table 2). In patients ≥60 years old, vaccination rates were significantly higher compared to patients <60 years old for all vaccines (74.8 vs. 57.8%, odds ratio (OR) 2.167, 95% confidence interval (CI) 1.213–3.870, *p* = 0.008 for influenza, 62.6 vs. 26.5%, OR 4.639, 95% CI 2.555–8.422, *p* < 0.0001 for pneumococcal and 18.7 vs. 3.7%, OR 6.059, 95% CI 1.772–20.712, *p* = 0.001 for herpes zoster vaccination), which underlines the efficacy of the German STIKO recommendation prioritizing older patients (Table 3).

### 3.3. Vaccination Quality

All applied influenza vaccines were quadrivalent, and all patients with a herpes zoster vaccination received the subunit vaccine.

In 32 patients (29.4% of all pneumococcal-vaccinated patients), the pneumococcal vaccination had been administered more than five years ago and, therefore, was outdated. Only a small minority of patients (*n* = 7, 3.2%) received the recommended stepwise pneumococcal vaccination (13-valent pneumococcal conjugate vaccine (PCV13) followed by 23-valent pneumococcal polysaccharide vaccine (PPSV23) at least eight weeks after PCV13 [9]), see Table 2.

### 3.4. Bi- and Multivariate Analysis

Female patients were more likely to be vaccinated against Streptococcus pneumoniae (OR 1.797, 95% CI 1.007–3.204, *p* = 0.046), while vaccination rates against influenza and herpes zoster (HZ) did not differ between the sexes (see Table 3). Furthermore, patients under glucocorticoid treatment were more likely to have received a pneumococcal (OR 2.386, 95% CI 1.389–4.097, *p* = 0.001) and HZ (OR 4.125, 95% CI 1.608–10.584, *p* = 0.002) vaccination. Vaccination rates against Streptococcus pneumoniae, influenza or HZ were highest in patients who had also been immunized against one of the other pathogens (Table 3). Only two patients under Janus kinase inhibitor (JAKi) therapy were vaccinated against HZ.

After multivariate logistic regression analysis (Table 3), ages >60 years, female sex, glucocorticoid use and influenza vaccination all remained independently associated with a pneumococcal vaccination. Pneumococcal vaccination was associated with an increased likelihood to obtain influenza vaccination, while no other parameter had a significant influence in the logistic regression analysis. As for the HZ vaccination, glucocorticoid use and a preceding pneumococcal vaccination remained independently associated. Further details can be obtained from Table 3.

## 4. Discussion

Vaccination rates for influenza and Streptococcus pneumoniae were comparatively high in our cohort. Our previous investigations a few years ago showed a much lower coverage for both vaccines [1,2]. In RA patients, 33.2% had a pneumococcal vaccination and 53.2% had an influenza vaccination [2]. Vaccination rates were lower in SLE patients, with 20.6% having a pneumococcal vaccination and 48.5% having an influenza vaccination [1]. Other German investigators also reported comparably low vaccination rates: 11.5% for pneumococcal vaccinations in RA patients [11] and 45.2 and 32.2%, respectively, for influenza and pneumococcal vaccinations in SLE patients [12]. These low rates are comparable to investigations in Latin America [13]. A 2020 study from Canada shows similar or slightly higher vaccination rates compared to the German results a few years ago [1,2,11], but compared to our current findings, they are significantly behind (with influenza and pneumococcal vaccination rates in 48.5 and 42% of RA patients, respectively) [14]. A more recent Canadian investigation in patients with RA also reports a low influenza vaccination rate of 46% [15]. Interestingly, a study from Greece on influenza vaccination found a high vaccination rate of 83% in patients with AIIRDs [16]. The varying vaccination rates across different countries most likely reflect individual diversities in study design and the distinct healthcare system in the country of origin as well as cultural differences.

In this respect, the continuous efforts and patient education during our outpatient visits led to a substantial increase in coverage for both influenza and pneumococcal vaccination. Using techniques such as best practise alerts, vaccination rates could probably be increased further [17]. In addition, it is possible that the COVID-19 pandemic led to increased vaccination awareness and contributed to the high vaccination rates. The pandemic puts people with AIIRDs at an increased risk since they have a high susceptibility to infections. Therefore, vaccination against COVID-19 has been and is of utmost importance. This imperative is reinforced by the fact that certain disease-modifying drugs are able to particularly impair the humoral immune response [18]. Fortunately, vaccination rates against COVID-19 are high in patients with autoimmune inflammatory rheumatic diseases [19]. In this context, the influenza vaccine was promoted to reduce the burden of COVID-19 since the odds of testing positive for COVID-19 have been shown to be reduced by 24% in persons who received an influenza vaccination [20]. Emphasizing the possible impact of the pandemic on vaccination rates, the vaccination rates we report here are high in comparison to a recent (pre-COVID-19) German report with low influenza and pneumococcal vaccination rates of 20 and 30.4%, respectively, in RA patients [21].

Since invasive pneumococcal disease (IPD) rates are higher in AIIRD patients compared to healthy controls [22], and since SLE patients in particular have a more complicated disease course [23,24], vaccination rates have to be improved further. Importantly, protection against pneumococcal disease is particularly low in patients with SLE and SpA. Usually, these patients are younger than patients with RA. Their younger age most likely contributes to the insufficient coverage, since guidelines for the general population recommend vaccination against pneumococcal disease (and also influenza) only for people ≥ 60 years old [10]. This reasoning is supported by the higher rate of vaccination against Streptococcus pneumoniae in patients ≥ 60 years old. Of interest, women and glucocorticoid users were more likely to be vaccinated against IPD, but not influenza. The meaning of these findings remains elusive. While it can be speculated that women might be more aware of vaccinations, this would mean they are also more likely to be vaccinated against influenza—which is not the case. Since glucocorticoids contribute to the infectious risk of AIIRD patients in a dose-dependent manner [25,26], the higher odds for being vaccinated against IPD might reflect the recommendation of the attending rheumatologist or GP considering this risk. Accordingly, a relationship between glucocorticoid use (≥7.5 mg prednisone equivalent) and influenza vaccination in SLE patients was reported before [12].

Furthermore, current IPD protection among our patients is probably impaired because a significant number of vaccinations were outdated and were applied more than five years ago. In addition, only a minority of patients received the recommended stepwise vaccination [10] against Streptococcus pneumoniae. This stepwise approach (PCV13 followed by PPSV23 vaccine) has been shown to induce an augmented immune response in the general population [27,28]. Contrary to pneumococcal vaccination, influenza vaccination is much more common in clinical practice and better known among patients. These facts might explain the higher influenza vaccination rates.

The lowest vaccination rate was found for herpes zoster (13.1%). There are several explanations for this finding. First, the HZ subunit vaccine is rather new: it was approved in 2017 in the US and 2018 in the EU [7]. Second, there was a relevant supply shortage of the subunit vaccine between 2018 and 2020, leading to fewer vaccinations than would have been warranted [29]. Finally, the 2019 EULAR recommendation recommends HZ vaccination primarily for “patients at risk” [9], while the 2011 recommendation only states “may be considered” [30]. The more recent 2019 recommendation also states that the subunit vaccine might “replace the live vaccine in patients with AIIRD” [9]. Nevertheless, the vaccination rate improved clearly—a few years ago, none of the investigated AIIRD patients had a HZ vaccination [1,2]. At that time, HZ vaccination using the only available live vaccine was much more challenging since live vaccines in general should be avoided during immunosuppression [9,30].

Since the HZ risk is increased under JAKi treatment [26], we expected a high HZ coverage among these particular patients. Currently, due to the low numbers of patients with JAKi therapy being vaccinated against HZ, we cannot draw meaningful conclusions on that patient group.

## 5. Conclusions

Vaccination rates for influenza and Streptococcus pneumoniae have improved during recent years. Besides the continuous efforts and patient education during our outpatient visits, the COVID-19 pandemic might have contributed to the high vaccination rates. Nevertheless, due to the high incidence and mortality of both diseases in patients with AIIRDs, the vaccination rates have to be increased further, particularly in SLE patients. Regarding HZ, vaccination rates have undoubtedly increased. However, at only 13.1%, vaccination coverage has to be improved further, particularly in patients at risk (e.g., under JAKi treatment).

## Figures and Tables

**Table 1:** Clinical characteristics of the included patients.

Variables	All Patients (*n* = 222)	RA (*n* = 97)	SpA ^1^ (*n* = 55)	SLE (*n* = 41)	Others ^2^ (*n* = 29)
female, *n* (%)	153 (68.9)	73 (75.3)	28 (50.9)	36 (87.8)	16 (55.2)
Mean age, years ± standard deviation	62.9 ± 13.9	67.5 ± 13.1	57.6 ± 12.3	56.5 ± 13.1	66.8 ±13.5
Patients ≥60 years, *n* (%)	139 (62.6)	75 (77.3)	23 (46)	17 (41.5)	21 (72.4)
Use of csDMARDs, *n* (%)	146 (66.1)	84 (86.6)	43 (86)	33 (82.5)	20 (69)
Use of bDMARDs, *n* (%)	104 (46.9)	55 (56.7)	39 (78)	4 (9.8)	7 (24.1)
Glucocorticoid use, *n* (%)	116 (52.5)	60 (61.9)	9 (18)	25 (62.5)	20 (69)

^1^—including axial spondyloarthritis and psoriatic arthritis; ^2^—including vasculitis, adult-onset still’s disease and connective tissue diseases other than SLE; bDMARD—biologic disease-modifying anti-rheumatic drug; csDMARD—conventional synthetic disease-modifying anti-rheumatic drug.

**Table 2:** Vaccination coverage and details.

Variables	All Patients (*n* = 222)	RA (*n* = 97)	SpA ^1^ (*n* = 55)	SLE (*n* = 41)	Others ^2^ (*n* = 29)
Influenza vaccination 2020, *n* (%)	152 (68.5)	69 (71.1)	37 (67.3)	26 (63.4)	20 (69)
Pneumococcal vaccination (any date), *n* (%)	109 (49.1)	58 (59.8)	20 (36.4)	14 (34.1)	17 (58.6)
Pneumococcal vaccination within the last 5 years, *n* (%)	77 (34.7)	43 (44.3)	13 (23.6)	11 (26.8)	10 (34.5)
Stepwise pneumococcal vaccination (PCV13 followed by PPSV23), *n* (%)	7 (3.2)	4 (4.1)	0 (0)	2 (4.9)	1 (3.5)
Herpes zoster vaccination, *n* (%)	29 (13.1)	17 (17.7)	5 (9.1)	2 (4.9)	5 (17.2)

^1^—including axial spondyloarthritis and psoriatic arthritis; ^2^—including vasculitis, adult-onset still’s disease and CTDs other than SLE; PCV13—13-valent pneumococcal conjugate vaccine; PPSV23—23-valent pneumococcal polysaccharide vaccine

**Table 3:** Bivariate and multivariate logistic regression analysis of the association between vaccination status and the patients’ characteristics.

	Bivariate Analysis		Multivariate Logistic Regression	
Vaccination	OR (95% CI)	*p* Value	OR (95% CI)	*p* Value
Streptococcus pneumoniae				
aged ≥ 60 years	4.639 (2.555–8.422)	<0.0001	5.071 (2.435–10.561)	<0.0001
female sex	1.797 (1.007–3.204)	0.046	2.679 (1.268–5.661)	0.01
csDMARD	1.382 (0.790–2.418)	0.257	0.956 (0.432–2.115)	0.911
bDMARD	0.927 (0.547–1.571)	0.779	1.421 (0.668–3.019)	0.361
Glucocorticoids	2.386 (1.389–4.097)	0.001	2.252 (1.171–4.330)	0.015
Influenza vaccination	9.734 (4.718–20.085)	<0.0001	10.552 (4.731–23.401)	<0.0001
Influenza				
aged >60 years	2.167 (1.213–3.870)	0.008	0.964 (0.478–1.944)	0.918
female sex	1.128 (0.615–2.069)	0.698	0.795 (0.391–1.616)	0.526
csDMARD	1.271 (0.702–2.302)	0.428	1.455 (0.648–3.265)	0.363
bDMARD	0.741 (0.420–1.308)	0.301	0.545 (0.252–1.181)	0.124
Glucocorticoids	1.312 (0.742–2.321)	0.350	0.821 (0.422–1.600)	0.563
Pneumococcal vaccination	9.734 (4.718–20.085)	<0.0001	10.347 (4.668–22.934)	<0.0001
Herpes zoster				
aged ≥60 years	6.059 (1.772–20.712)	0.001	2.558 (0.672–9.735)	0.168
female sex	1.193 (0.5–2.847)	0.690	0.817 (0.300–2.220)	0.691
csDMARD	2.168 (0.842–5.582)	0.102	2.464 (0.818–7.418)	0.109
bDMARD	0.751 (0.341–1.658)	0.478	0.545 (0.212–1.401)	0.208
Glucocorticoids	4.125 (1.608–10.584)	0.002	3.209 (1.178–8.740)	0.023
Influenza vaccination	3.199 (1.068–9.581)	0.03	1.206 (0.336–4.334)	0.774
Pneumococcal vaccination	8.036 (2.693–23.980)	<0.0001	4.723 (1.304–17.109)	0.018

bDMARD—biologic disease-modifying anti-rheumatic drug; CI—confidence interval; csDMARD—conventional synthetic disease-modifying anti-rheumatic drug; OR—odds ratio.

## Data Availability

The data presented in this study are available on reasonable request from the corresponding author. The data are not publicly available due to privacy reasons.
